# Perioperative Lung Ultrasound Findings in Elective Intra-Abdominal Surgery: Associations with Postoperative Pulmonary Complications

**DOI:** 10.3390/jcm13237098

**Published:** 2024-11-24

**Authors:** Moshe Rucham, Yotam Lior, Lior Fuchs, Benjamin F. Gruenbaum, Asaf Acker, Alexander Zlotnik, Evgeni Brotfain

**Affiliations:** 1 Division of Anesthesiology and Critical Care, Soroka University Medical Center, Beer Sheva 8453227, Israel; mosherucham@gmail.com (M.R.); alekszl@clalit.org.il (A.Z.); 2 Faculty of Health Science, Ben-Gurion University of the Negev, Beer Sheva 8410501, Israel; liorfuchs@gmail.com (L.F.);; 3 Division of Anesthesia, Intensive Care, and Pain Management, Tel Aviv Medical Center, Tel Aviv 6777801, Israel; matoyster@gmail.com; 4 Faculty of Medicine, Tel Aviv University, Tel Aviv 6997801, Israel; 5 Medical Intensive Care Unit, Department of Internal Disease, Soroka University Medical Center, Beer Sheva 8410501, Israel; 6 Department of Anesthesiology and Perioperative Medicine, Mayo Clinic, Jacksonville, FL 32224, USA; gruenbaum.benjamin@mayo.edu; 7 Department of Orthopedic Surgery, Soroka University Medical Center, Beer Sheva 8453227, Israel

**Keywords:** lung ultrasound, lung ultrasound score, point-of-care ultrasonography, postoperative pulmonary complications

## Abstract

**Background:** For patients undergoing abdominal surgery, postoperative pulmonary complications (PPCs) are a major source of morbidity and mortality. The use of point-of-care ultrasonography (POCUS), and specifically POCUS of the lungs, has seen many advancements in recent years. **Objectives:** We hypothesize that perioperative lung ultrasonography can be used as a predictor for PPCs. **Methods:** In a Single, 1000 beds, trauma level I medical center, patients presenting for elective intra-abdominal surgery with no severe pulmonary or cardiac diseases were evaluated preoperatively with a standardized 12-point lung ultrasound exam. A second identical exam was performed after surgery in the post-anesthesia care unit. PPCs were also documented. All lung ultrasound exams were presented to a blinded researcher and a lung ultrasound score (LUS) was calculated. Statistical analysis comparing pre- and postoperative LUS and PPC scores were performed. **Results:** A total of 61 patients were evaluated. The pre-surgery median LUS was 0 (in the range of 0–6) and the post-surgery median LUS was 3 (in the range of 0–14). The pre- to postsurgical LUS delta was 3.4 (standard deviation of 3.3). A postoperative LUS of 6 or more was defined as “high.” A High LUS did not correlate with prolonged post-anesthesia care unit or hospital stay, prolonged oxygen support, or number of desaturation events. **Conclusion:** For elective abdominal surgery in relatively healthy patients, preoperative LUS usually begins at a normal level and becomes worse after general anesthesia. However, this difference in LUS is not significantly associated with clinically relevant postoperative pulmonary complications such as prolonged oxygen therapy, pneumonia, and noninvasive or invasive mechanical ventilation. Trial registration: Clinicaltrials.gov identifier: NCT05502926. Summary: This paper explores the use of point-of-care ultrasonography as a predictor for postoperative pulmonary complications. The findings suggest that while the lung ultrasound score worsens with general anesthesia, the differences are not significantly associated with postoperative pulmonary complications.

## 1. Introduction

Postoperative pulmonary complications (PPCs) are lung pathologies that manifest after surgery. The exact definitions and diagnoses of these pathologies vary, but usually include respiratory failure, the need for postoperative invasive or non-invasive positive pressure ventilation, and the occurrence of pulmonary infection, including pneumonia, tracheobronchitis, atelectasis, pleural effusion, bronchospasm, acute respiratory distress syndrome (ARDS), pulmonary edema, and aspiration pneumonitis [1].

PPCs are a major source of morbidity and mortality postoperatively, with a mortality rate of 14–30% in patients with PCCs compared to 0.2–3% in patients without PCCs [2]. The ability to predict which patients are at greatest risk for PCCs is limited, despite many risk stratification tools that are available [3,4,5]. These tools mainly consider baseline pulmonary diseases and surgical parameters such as surgery location, length, and emergency surgery. Preoperative assessment of the patient is mostly limited to pre-induction pulse oximetry saturation.

With the increasing availability of portable ultrasound machines, the utilization of point-of-care ultrasound (POCUS) has garnered significant attention as an intriguing new tool for the efficient evaluation of patients at their bedside. The lung, long excluded from ultrasound, has posed challenges due to the fact that a healthy lung is air filled and not ultrasound compatible, and the artifacts generated by air-filled cavities complicate results. However, new understanding of these artifacts has allowed for multiple uses in diagnosing lung pathologies, such as pneumothorax, pleural effusion, atelectasis, and pulmonary edema. Lung ultrasound has been shown to successfully predict the severity of pulmonary disease compared to computerized tomography [6] and is more accurate in diagnosing PPCs when compared to traditional physical exams and chest X-rays [7].

The use of a cumulative score while assessing the lungs, referred to as the lung ultrasound score (LUS), has been described in the literature [6,7,8,9]. The term is poorly standardized, though, with different studies using different scoring systems. All use a bilateral approach, dividing the lungs into zones, with 4 to 9 zones on each side. Each zone is assessed by ultrasound and given a score. A score of 0 represents normal or near-normal lung appearance and higher numbers represent pathologies: multiple B-lines, loss of aeration, and frank atelectasis. This grading system is not standardized. A higher LUS has been shown to predict poorer postoperative oxygenation among patients undergoing scoliosis corrective surgery and laparoscopic surgery [8,9] and prolonged stay and ventilator time among surgical patients admitted to the ICU postoperatively [10].

Canty et al. [11] have shown that in emergency non-cardiac surgery, bedside echocardiography given before anesthesia caused a major change in the anesthesia plan in 44% of the cases, including changing to a more invasive hemodynamic monitoring technique, planning for postoperative ICU admission, changing of planned surgery, or delaying surgery for further cardiac evaluation. In 30 patients undergoing elective abdominal laparoscopic procedures, Monastesse, et al. [9] have shown that sequential LUS exams pre-induction, post-induction, after pneumo-peritoneum, on admission to the recovery unit, and on discharge from the recovery unit demonstrate higher LUS correlated with lower P/F ratio (a measurement of lung function) in the recovery unit. The aim of our study was to investigate the association between preoperative and postoperative LUS and PPCs among elective abdominal surgery patients.

## 2. Ethics

This study was approved and supervised by the Human Research and Ethics Committee at the Soroka Medical Center located at 151 Rager street, Beer Sheva Israel starting on 1 March 2022, chaired by Prof Eitan Lonnenfeld (RN 10-22-SOR).

## 3. Methods

This study was conducted at the Soroka Medical Center, a 1000-bed, trauma level I, university teaching hospital located in Beer Sheva in southern Israel. We recruited patients undergoing elective abdominal surgery at Soroka between April 2022 and September 2022. The clinical data were extracted from a computerized clinical information system (OFEK iMDsoft^®^, Tel Aviv, Israel).

During visits to the pre-surgery clinic, patients were screened for inclusion and exclusion criteria. Those who were eligible for the study were asked to give formal consent to participate in the study. After consent, the examiner reviewed the patient’s past medical history and surgery plan.

### 3.1. Inclusion Criteria

Included patients were 18 years of age or older, with ASA class I, II, or III, presenting for elective intra-abdominal surgery under general anesthesia who were not planned for long-term postsurgical mechanical ventilation, and who had no past medical history of cardiovascular or respiratory issues.

### 3.2. Exclusion Criteria

We excluded patients who had pre-existing moderate or severe heart or lung disease, a history of ischemic heart disease, moderate or severe systolic or diastolic heart failure, moderate or severe pulmonary hypertension, or moderate or severe obstructive or interstitial lung disease. Patients were also excluded if they had a severe intraoperative pulmonary complication (laryngospasm, bronchospasm, anaphylaxis, emergency surgical airway), required unplanned long-term mechanical ventilation, or died during surgery.

### 3.3. Study Protocol

**Figure 1:**
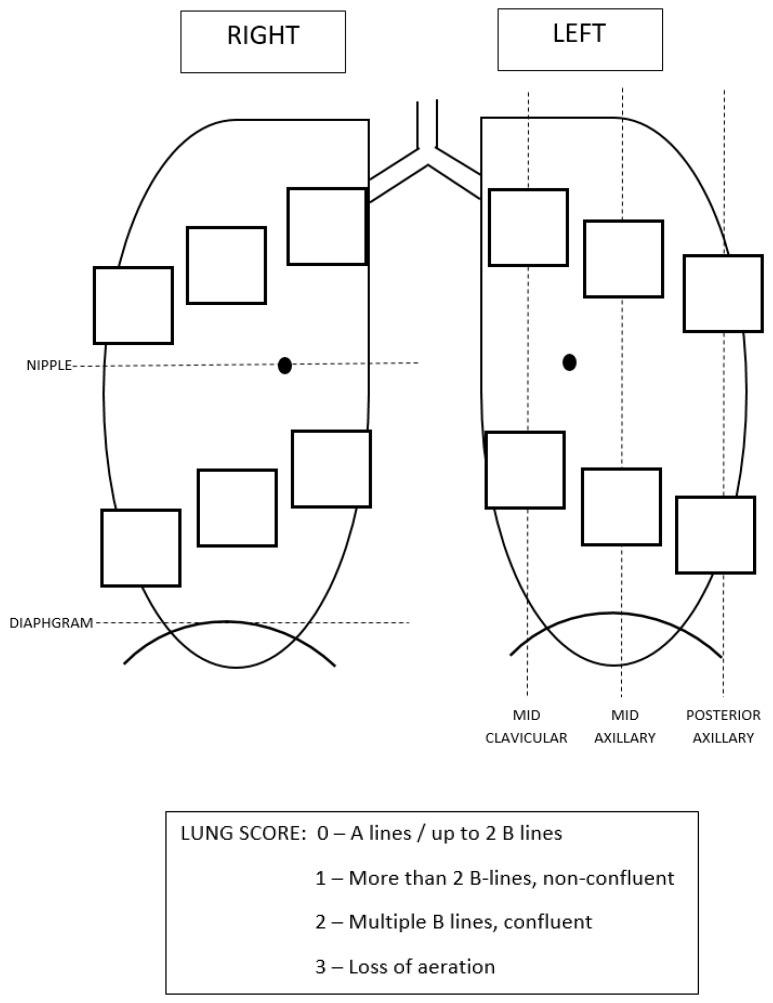
POCUS score: anatomical sites. A total of 12 exam points per patient were recorded, 6 for each side.

**Figure 2:**
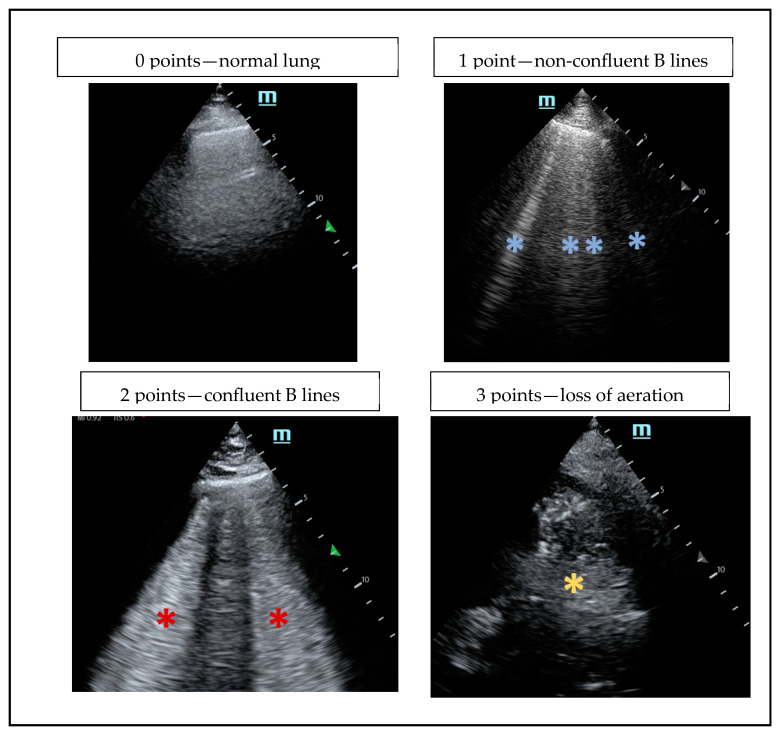
POCUS score: US views. Blue *—non-confluent B lines. Red *—confluent B lines. Yellow *—loss of aeration.

On the day of surgery, the preoperative LUS exam was performed at the pre-surgery unit admission area (Figure 1 and Figure 2).

The ultrasound exams were administered using a portable POCUS machine (Mindray TE7, Shenzhen, China). The examiner used a phased array transducer (Mindray, P4-2s) in the “lung” preset. For deeper structures (lung consolidation or pleural effusion) or in patients with obesity, the “adult cardiac” preset was used, per the examiner’s preference. When the pleura was investigated, the examiner could also use a linear array transducer (Mindray, L12-4s) using the “lung” preset. On nine occasions, due to technical problems, the phased array transducer was unavailable, and a convex array probe (Mindray, C5-2s) on the “lung” preset was used. Exams were saved digitally with identifying information removed.

For all of the preoperative and postoperative exams, the transducer was aligned in a sagittal position, and the exam was recorded in 6 zones in each side (Figure 1). The nipple line, diaphragm, midclavicular, mid-axillary, and posterior axillary lines were used to determine the correct borders for the different zones (Figure 1).

After surgery, the examiner reviewed the electronic anesthetic record and performed the postoperative LUS exam within 30 min of arrival at the recovery unit. Upon discharge from the recovery unit, the examiner reviewed the recovery unit’s electronic record. After discharge from the hospital, the examiner reviewed the patient’s postoperative surgical ward electronic record. All LUS exams were recorded and reviewed, deidentified, and blinded to the time recorded (pre- or post-surgery) by an LUS POCUS expert with over ten years of experience. This examiner was also blinded to all demographic information and clinical data of the patients. The expert was asked to score the level of pathology of the lung exams by using the following scoring system: a score of 0 was given for a normal lung field showing A-lines or having few (≤2) B-lines; a score of 1 was given for lung fields showing more than 2 B-lines and non-confluent; a score of 2 was given for lung fields showing multiple, confluent B-lines; and a score of 3 was given if the lung field showed signs of consolidation. The maximal possible score was 36. This scoring system has been well documented in previous studies [6,8,9,10].

The co-primary endpoint was the occurrence of any of the following: pneumonia, unplanned invasive or non-invasive ventilation, ICU admission, or death. Secondary endpoints included prolonged postoperative oxygen therapy (more than 12 h postoperatively), desaturation events, and hospital stay.

### 3.4. Variables and Measures

Baseline data were collected from the hospital’s electronic recording system. We documented patients’ demographic data (age, gender, weight, height, BMI, ASA score, history of smoking, asthma, hypertension, and diabetes) and the type of surgery planned.

The anesthesia data included the type of surgical approach, ventilation mode, anesthetic agents, paralytic administration, administered FiO_2_, tidal volumes, respiratory rate, positive end-expiratory pressure (PEEP), anesthesia duration, and method of extubation. For dynamic ventilator settings such as FiO_2_, tidal volumes, respiratory rate, and PEEP—which can change multiple times during the intraoperative period—the most prevalent setting (the one used for the majority of the anesthesia duration) was recorded. This approach was chosen due to the limited statistical power to analyze multiple intraoperative changes across these factors.

The post-anesthesia care unit (PACU) data included the level of consciousness on admission, admission oxygen support, admission saturation, oxygen face mask total time, oxygen nasal cannula total time, need for supportive ventilation, bronchodilator treatment, total recovery time, discharge saturation, and discharge oxygen support.

The surgical ward data included the need for oxygen support or supportive ventilation up to 48 h postoperatively, re-intubation or ICU admission, desaturation events, pathologic chest X-ray, diagnosis of pneumonia, the total length of hospital stay, and death.

Data collected in this study were documented using summary tables. Continuous variables with normal distributions are presented as means and standard deviations, while ordinary variables or continuous variables with non-normal distributions are presented as medians with interquartile ranges (IQRs). Categorical variables are presented as counts and percentages of the total. Given the size of the groups in the cohort, a conservative approach was applied when comparing continuous or ordinal variables by using the non-parametric Mann–Whitney test. Categorical variables were tested using Pearson’s χ2 test for contingency tables or Fisher’s exact test, as appropriate. All statistical tests were performed at α = 0.05 (2-sided). Data were analyzed using the IBM SPSS 27 Statistics software.

## 4. Results

**Figure 3:**
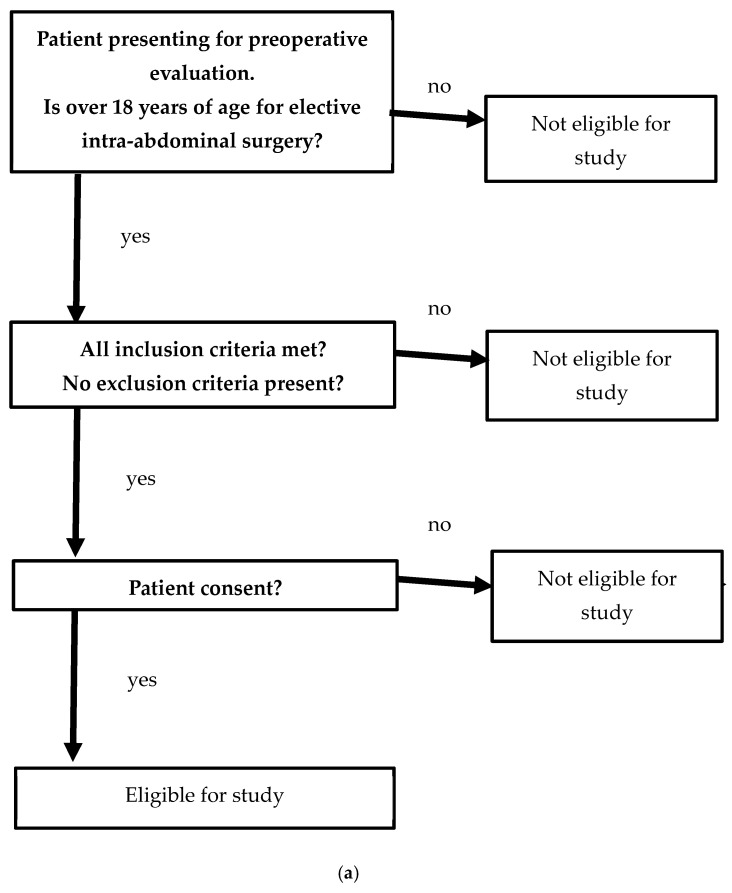

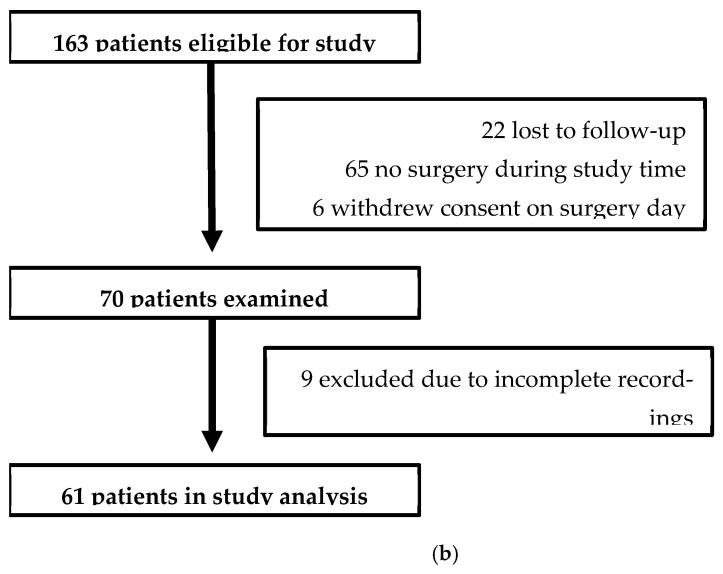
(**a**) Patient recruitment. (**b**) Study design.

Overall, 163 patients were recruited into this study. Of these patients, 22 were lost to follow-up (surgery was performed while no investigator was on site), 65 did not have surgery performed during the study time, and 6 withdrew consent on the day of surgery (Figure 1). A total of 70 patients had LUS recorded. Of these, nine were excluded due to incomplete data (no postoperative LUS recorded). A total of 61 patients were eligible for statistical analysis (Figure 3). The blinded reviewer analyzed a total of 1392 clips; out of them, 14 (1%) were considered to have poor technical quality and were excluded from the final analysis.

Due to the small sample size, a multivariate analysis was not feasible. No patient had a preoperative LUS score that was considered high (defined as equal to or higher than 6 points), so the statistical analysis was performed only on the postsurgical exam. A score equal to or above 6 was considered pathological (Figure 2).

Patients’ baseline characteristics and comorbidities, as well as type and duration of anesthesia and surgery were not statistically significantly associated with a pathological postoperative LUS (Table 1 and Table 2).

Out of 61 patients in the data analysis, 55 had a preoperative LUS exam performed and recorded. A total of 45 out of 55 patients (81%) had an LUS of 0 preoperatively, and the highest preoperative LUS was 6, recorded in a single patient. In the postoperative exam, 13 out of 61 patients (21%) had an LUS of 0. The highest LUS recorded was 14. On average, the LUS increased by 3.4 points from the preoperative to postoperative exam (Table 3).

A total of 22 patients met the criterion for a high LUS score (6 or higher). Patients with high LUS did not spend more time in PACU or need more oxygen. There were no instances of re-intubation, unplanned ICU admission, or need for non-invasive positive pressure ventilation during their PACU stay.

In the late postoperative period, no patient needed re-intubation, unplanned ICU admission, or non-invasive positive pressure ventilation during a stay in the surgical ward. There were also no instances of pneumonia or death. Five patients needed oxygen support via nasal cannula for over 12 h after surgery, and seven had desaturation (pulse oxygen saturation < 90%) recorded. Of these seven, almost all had a preoperative LUS score of 0 (one had no preoperative LUS exam performed). Four had a postoperative LUS that was low (range: 1–2 points), and three had a high postoperative LUS (range: 6–7 points). Three had a postoperative chest X-ray (CXR) performed. One patient, undergoing a partial hepatectomy, had a CXR on postoperative day (POD) 2 that showed a small right-sided pulmonary effusion. This patient had a postoperative LUS score of 6 points. Another patient, also undergoing a partial hepatectomy, had a CXR on POD 2 that showed medium-sized bilateral pulmonary effusions. This patient had a postoperative LUS score of 6 points. The last patient underwent an exploratory laparotomy that developed into a bilateral salpingo-oophorectomy. She had a CXR on POD 0 that showed bilateral perihilar consolidation. This patient had a postoperative LUS score of 1. A high LUS was not associated with these postoperative pulmonary complications. The total hospital length of stay was also not significantly different among the groups (Table 4).

Intraoperative ventilation settings were compared to postoperative consolidations on the POCUS exam. Consolidations (in any lung zone) were divided into small (under 4 cm) or large (4 cm or larger). A total of 36 patients (59%) had any consolidations on the postoperative exam. Of these, 29 presented with small (48%) and 7 with large (11%) consolidations. These new consolidations represent postoperative atelectasis and loss of lung volume, a common mechanism for postoperative pulmonary complication. No statistical difference was found between the most prevalent ventilator settings during surgery (respiratory rate, total tidal volume, tidal volume per kg, FiO_2_, and PEEP) and the detection of consolidations on the postoperative exam. Patient demographics, medical history, and type and length of surgery and anesthesia were also non-significant (Table 5).

## 5. Discussion

As PPCs can be a major surgical complication, our study investigated the use of lung point-of-care sonography (POCUS) for the early detection of elective surgery patients at risk for PPCs. Our study focused on abdominal surgery, which has been reported to correlate with a higher risk of PCCs. We included patients with ASA status > 1 but with no cardiac or pulmonary comorbidities, all known additional risk factors for PCCs [12]. To our knowledge, our study is the first non-ICU study to investigate PPCs beyond the immediate postoperative period and is the only one that used a blinded reader (to the PPCs and to the pre- and post-surgery status) as part of its methodology.

Changes in lung parenchyma during general anesthesia have been observed with lung POCUS in many studies. These changes were noted in high-risk surgeries such as open heart and scoliosis repair [7,8] as well as medium and high-risk surgeries such as hysteroscopies, laparoscopies, and laparotomies [13]. In one study, Krawczyk et al. showed similar results as ours, with patients undergoing general anesthesia showing worse LUS with prominent B-lines and loss of aeration after surgery. That study also mentioned that no PPCs occurred in its patients, but did not provide details on which PPCs were recorded and in what time frame [13].

As lung ultrasound increases in use in perioperative medicine, it is crucial to investigate its role not only as an indicator of lung mechanics but also as a predictive risk stratification tool for the postoperative period. In this regard, our study clearly showed the worsening of lung mechanics postoperatively, but, more importantly, showed that this finding does not correlate to worse postsurgical outcomes or clinically relevant postoperative pulmonary complications, such as prolonged oxygen therapy, pneumonia, and noninvasive or invasive mechanical ventilation, in a relatively healthy, elective intra-abdominal surgery population.

Jakobson et al. [14] showed that LUS could be more effective than chest X-ray imaging in the detection of PPCs after thoracic surgery, a type of surgery where postoperative screening with chest X-ray is widely accepted. In a multi-center study, Ramsingh et al. [15] showed that in patients suffering from desaturation in the postoperative period, POCUS was useful in rapid diagnosis as it reduced the number of suspected diagnoses from a median of 5 pre-exam to a median of 1 post-exam. The study also showed that the use of POCUS significantly reduced the time spent in the PACU (120 vs. 96 min).

LUS provides a rapid, cost-effective, and safe alternative for postoperative evaluation. However, it is not without limitations. Ultrasonography is operator-dependent, and thus formal training, practice, and supervision are mandated. Recent guidelines on the use of POCUS for the diagnosis of pulmonary diseases [16] emphasize the need for training clinicians who use POCUS in the use and interpretation of findings and ongoing care quality assessment. Although POCUS has been shown to increase the accuracy of diagnosing pulmonary pathologies by up to 32% more than traditional modalities, it can have a high false positive rate. It may not be realistic to implement routine LUS exams for all postoperative patients, regardless of pre-test predictive factors. The European Society of Anesthesiology suggests using a POCUS examination of the heart and lung by a trained anesthetist in selected patients with concerns regarding cardiovascular comorbidity before urgent or emergency surgery to address significant cardiac abnormalities, these suggestions were not shown to reduce morbidity or mortality in any patient population [17]. Furthermore, POCUS examinations pose no risk to patients. When performed by an anesthesiologist, they do not delay surgery with time-consuming exams or consultations. Additionally, with the widespread availability of ultrasound machines, the operating costs associated with these exams are minimal.

The optimal timing for the LUS exam as a prognostic factor is also unknown. Our study protocol dictated that the postoperative LUS exam be performed within 30 min of the patient’s arrival to the PACU. Some patients were awake during the exam, some were still deeply sedated, and some were mildly sedated. All of the exams were performed in the PACU. Studies show that the functional residual capacity diminishes postoperatively and reaches its lowest point on the second postoperative day [18], suggesting that the exam in the PACU might not be the best predictor, but rather could take place in the surgical ward on the first or second postoperative day. If feasible, we believe that further research should focus on LUS exams on arrival to the surgical ward, or even the next day after surgery.

At this time, there is no clear prognostic value for the LUS postoperatively. In our study, some patients had a significant increase in postoperative LUS but did not have any early or late PPCs and had an otherwise routine postoperative recovery. With no clear prognosis and risk stratification strategy, some patients may be categorized as high risk per the LUS exam, and extensive resources would be directed (monitored bed, CT, and other radiological studies) to the patient with no clear benefit and, in the case of radiation exposure, may cause harm. For these reasons, it is crucial to further investigate which patient populations, and with which surgeries, will benefit from routine postoperative lung ultrasonography exams. Our results suggest that LUS examinations, conducted immediately preoperatively and postoperatively, have limited prognostic value in a generally healthy population. Future studies should explore the potential utility of LUS in patients with significant comorbidities and at later time points following surgery.

Our study had some major limitations, including its small sample size. Ethical concerns mandated that patients could not be enrolled on the day of surgery. Due to this, enrollment was performed at the pre-surgery clinic, when the date and time of the surgery were still unknown. As a result, more than half of the patients recruited were lost to follow-up because they went into surgery without an LUS exam or did not have their surgery during the study’s allocated time frame. With a larger sample size, more statistically significant results may have been found, and a multivariate analysis may have been performed. Our study population consisted of fairly healthy (ASA I-III) patients with no severe pulmonary or cardiac comorbidities, undergoing elective intra-abdominal surgery. In this population, we found no severe postoperative pulmonary complications, and non-severe complications such as prolonged oxygen therapy were not frequent. It is possible that the LUS exam would be more predictive of complications in a population more prone to pulmonary complications, such as in patients admitted for emergency surgery or those with pulmonary or cardiac comorbidities. Our relatively healthy population with a low risk of major complications, coupled with our relatively small sample size, is one of the biggest weaknesses of our study. Nevertheless, we believe that this baseline data are useful in describing the relationship between postoperative LUS and postoperative complications, as our study is the first to include both late postoperative complications and a blinded examiner. These findings serve as the basis for further research in a larger set of patients, with more comorbidities and more diverse surgeries. Thus, we are currently developing a follow-up study to address these questions.

Our data show that for relatively healthy patients undergoing elective abdominal surgery, preoperative LUS is typically within normal limits. After general anesthesia, the LUS became significantly worse, but the higher LUS was not significantly associated with postoperative complications. Postoperative pulmonary complications were uncommon and mild in our cohort, even among patients with high LUS. It is possible that in a larger cohort, including patients with more comorbidities or undergoing non-elective surgery, the LUS will be a helpful tool in identifying those at risk for severe PPCs. Further prospective studies are needed with a patient population that includes obese patients, non-elective surgeries, and patients undergoing other surgeries. Identifying a patient population that is particularly at risk of PPCs, and correlated with bad outcomes, is of critical importance to clinicians performing high- and medium-risk surgeries on these patients. The relationship between LUS scores and PPCs in a wide variety of patient populations and surgeries remains a challenge, and more research is necessary to identify best practices for predicting and improving patient outcomes.

## Figures and Tables

**Table 1:** Cohort baseline characteristics.

Variable		All Population (*n* = 61)	Postsurgical Score		*p*-Value
			<6 (n = 39)	≥6 (n = 22)	
Age, mean (SD), y		52.4 (15.7)	53.4 (17)	50.6 (13.4)	0.5
Gender, male, n (%)		29 (47.5)	20 (51.3)	9 (40.9)	0.44
BMI, mean (SD)		30.2 (24.5)	30 (8.5)	30.6 (7.1)	0.45
Obesity, n (%)		26 (42.6)	17 (43.6)	9 (40.9)	0.84
ASA, n (%)	1–2	45 (73.8)	29 (74.4)	16 (72.7)	0.89
	3	16 (26.2)	10 (25.6)	6 (27.3)	
Smoker, n (%)		16 (26.2)	11 (28.2)	5 (22.7)	0.64
Comorbidities, n (%)	DM	7 (11.5)	4 (10.3)	3 (13.6)	0.7
	HTN	18 (29.5)	13 (33.3)	5 (22.7)	0.38
	Asthma	4 (6.6)	3 (7.7)	1 (4.5)	1

SD—standard deviation, BMI—body mass index, ASA—American society of anesthesiologists physical status, DM—diabetes mellitus, HTN—hypertension.

**Table 2:** Surgery and anesthesia.

Variable		All Population (n = 61)	Postsurgical Score		*p*-Value
			<6 (n = 39)	≥6 (n = 22)	
Surgery, n (%)	Hernia	22 (36.1)	12 (30.8)	10 (45.5)	n/a
	Colectomy	6 (9.8)	4 (10.3)	2 (9.1)	
	Cholecystectomy	10 (16.4)	10 (25.6)	0 (0)	
	Other	23 (37.7)	13 (33.3)	10 (45.5)	
Surgical approach, n (%)	Open	10 (16.4)	6 (15.4)	4 (18.2)	n/a
	Laparoscopic	44 (72.1)	27 (69.2)	17 (77.3)	
	Both	7 (11.5)	6 (15.4)	1 (4.5)	
Ventilation, n (%)	ET	58 (95.1)	36 (92.3)	22 (100)	0.55
	LMA	3 (4.9)	3 (7.7)	0 (0)	
Anesthesia, n (%)	Volatile gas	60 (98.4)	38 (97.4)	22 (100)	0.45
	TIVA	1 (1.6)	1 (2.6)	0 (0)	
Anesthesia length, median (IQR), min		70 (50–107)	65 (50–105)	70 (50–110)	0.77
Paralysis, n (%)		58 (95.1)	37 (94.9)	21 (95.5)	0.92
Extubation, n (%)	Awake	27 (44.3)	19 (48.7)	8 (36.4)	0.35
	Deep	34 (55.7)	20 (51.3)	14 (63.6)	

ET—endotracheal tube, LMA—laryngeal mask airway, TIVA—total intravenous anesthesia, IQR—interquartile range.

**Table 3:** Pre- and postsurgical US findings.

Variable	All Population (n = 61)	Postsurgical Score		*p*-Value
		<6 (n = 39)	≥6 (n = 22)	
Presurgical score, median (IQR)	0 (0–0)	0 (0–0)	0 (0–0)	0.73
Postsurgical score, median (IQR)	3 (1–6)	2 (0–3)	6.5 (6–8.2)	n/a
Pre-postsurgical score delta, mean (SD)	3.4 (3.3)	1 (0–3)	6 (6–8)	n/a

IQR—interquartile range, SD—standard deviation.

**Table 4:** Outcomes.

Variable		All Population (n = 61)	Postsurgical Score		*p*-Value
			<6 (n = 39)	≥6 (n = 22)	
PACU responsiveness at arrival, n (%)	Alert	10 (16.4)	6 (15.4)	4 (18.2)	n/a
	Vocal	18 (29.5)	11 (28.2)	7 (31.8)	
	Pain	10 (16.4)	6 (15.4)	4 (18.2)	
	Unresponsive	23 (37.7)	16 (41)	7 (31.8)	
PACU admission O_2_ supply, n (%)	Room air	2 (3.3)	2 (5.1)	0 (0)	0.53
	Face mask	59 (96.7)	37 (94.9)	22 (100)	
PACU discharge O_2_ support, n (%)	Room air	29 (47.5)	21 (53.8)	8 (36.4)	n/a
	Nasal cannula	25 (41)	14 (35.9)	11 (50)	
	Face mask	7 (11.5)	4 (10.3)	3 (13.6)	
PACU admission O_2_ saturation, mean (SD), %		98.4 (3.4)	98.5 (3.8)	98.4 (2.7)	0.11
PACU time with face mask, median (IQR), minutes		40 (20–60)	40 (20–50)	42 (20–70)	0.26
PACU time with nasal cannula, median, (IQR), minutes		0 (0–37.5)	0 (0–30)	5 (0–40)	0.27
Total PACU O_2_ enrichment time, median (IQR), minutes		60 (20–90)	50 (20–80)	60 (40–110)	0.62
Total PACU time, median (IQR), minutes		80 (65–110)	80 (65–100)	85 (67.5–110)	0.31
PACU discharge O_2_ saturation, mean (SD), %		97.6 (2.3)	97.4 (2.6)	98.1 (1.8)	0.4
Ward nasal cannula > 12 h, n (%)		5 (8.2)	2 (5.1)	3 (13.6)	0.57
Saturation < 90%, n (%)		7 (11.5)	4 (10.3)	3 (13.6)	0.7
Physical therapy, n (%)		13 (21.3)	8 (20.5)	5 (22.7)	1
Length of hospital stay, median (IQR), days		3 (3–3.5)	3 (3–3)	3 (3–5.25)	0.17

PACU—post anesthesia care unit, IQR—interquartile range, SD—standard deviation.

**Table 5:** Intraoperative ventilation and postoperative consolidations.

Variable		All Population (n = 61)	No Consolidations (n = 25)	Consolidations (n = 36)	*p*-Value
Age, mean (SD), y		52.44 (15.76)	55.4 (16.58)	50.39 (15.05)	0.15
Gender, male, No. (%)		29 (47.5)	13 (52)	16 (44.4)	0.56
Weight, mean (SD), kg		84.41 (22.95)	80.84 (25.31)	86.89 (21.17)	0.21
Height, mean (SD), cm		166.93 (8.13)	167.88)	166.28 (7.55)	0.45
BMI, mean (SD)		30.27 (24.59)	28.5 (8.21)	31.51 (7.7)	0.57
Obesity, n, (%)		26 (42.6)	8 (32)	18 (50)	0.16
ASA, n (%)	1–2	45 (73.8)	20 (80)	25 (69.4)	0.36
	3	16 (26.2)	5 (20)	11 (30.6)	
ASA score, median (IQR)		2 (2–3)	2 (2–2)	2 (2–3)	0.051
Surgical approach, n (%)	Open	10 (16.4)	5 (20)	5 (13.9)	n/a
	Lap	44 (72.1)	16 (64)	28 (77.8)	
	Both	7 (11.5)	4 (16)	3 (8.3)	
Ventilation, n (%)	ET	58 (95.1)	23 (92)	35 (97.2)	n/a
	LMA	3 (4.9)	2 (8)	1 (2.8)	
Anesthesia, n (%)	Gas	60 (98.4)	24 (96)	36 (100)	n/a
	TIVA	1 (1.6)	1 (4)	0 (0)	
Paralytics, n (%)		58 (95.1)	23 (92)	35 (97.2)	0.56
Extubation, n (%)	Awake	27 (44.3)	10 (40)	17 (47.2)	0.58
	Deep	34 (55.7)	15 (60)	19 (52.8)	
Ventilatory parameters	FiO_2_, mean (SD) %	48.75 (9.6)	50.95 (7.68)	47.04 (10.67)	0.15
	TV, mean (SD), ml	493.15 (51.98)	491 (48.12)	494.81 (47.84)	0.9
	TV/kg, mean (SD)	6.33 (1.54)	6.64 (2.11)	6.1 (0.85)	0.51
	RR, median (IQR)	12 (12–14)	12 (12–13.5)	12 (12–14)	0.44
	PEEP	5.15 (1.1)	4.95 (0.22)	5.31 (1.44)	0.32
Length of anesthesia, median (IQR), min		70 (50–107.5)	70 (55–131.5)	65 (50–88.75)	0.48

IQR—interquartile range, SD—standard deviation, ET—endotracheal tube, LMA—laryngeal mask airway, TIVA—total intravenous anesthesia, RR—respiratory rate, PEEP—positive end expiratory pressure.

## Data Availability

The data that support the findings of this study are available from the corresponding author upon reasonable request.
